# Objectively Assessed Exercise Behavior in Chinese Patients with Early-Stage Cancer: A Predictor of Perceived Benefits, Communication with Doctors, Medical Coping Modes, Depression and Quality of Life

**DOI:** 10.1371/journal.pone.0169375

**Published:** 2017-01-10

**Authors:** Zhunzhun Liu, Lanfeng Zhang, Songsong Shi, Wenkai Xia

**Affiliations:** 1 Departments of Nursing, Jiangsu Jiangyin People’s Hospital, Jiangyin, Jiangsu, China; 2 Departments of Nursing, Nantong Tumor Hospital, Nantong, Jiangsu, China; 3 Faculty of Nursing, University of NanTong, Nantong, Jiangsu, China; 4 Departments of Nephrology, Jiangsu Jiangyin People’s Hospital, Jiangyin, Jiangsu, China; University of Rochester, UNITED STATES

## Abstract

This study sought to identify factors associated with objectively assessed exercise behavior in Chinese patients with early-stage cancer. Three hundred and fifty one cancer patients were recruited from the Affiliated Jiangyin Hospital of Southeast University Medical College and the Nantong Tumor Hospital. One-way ANOVA, Pearson Chi-square tests and regression analysis were employed to identify the correlations between physical exercise and the measured factors. The results showed that occupation type (*χ*^2^ = 14.065; p = 0.029), monthly individual monthly income level (*χ*^2^ = 24.795; p = 0.003), BMI (*χ*^2^ = 15.709; p = 0.015) and diagnosis (*χ*^2^ = 42.442; p **<** 0.000) were significantly correlated with the subjects self-reported exercise with different frequency per week. Differences in the frequency of exercise were associated with different degrees of reported Benefit Finding (BF) (F = 24.651; p **<** 0.000), communication with doctors (F = 15.285; p **<** 0.000), medical coping modes (F = 45.912; p **<** 0.000), social support (F = 2.938; p = 0.030), depression (F = 6.017; p **<** 0.000), and quality of life (F = 12.288; p **<** 0.000). Multiple regression analysis showed that 1.6%-6.4% of the variance in five variables, excluding social support and optimism could be explained by exercise. Our results indicated that benefit finding, medical coping modes, communication with doctors, social support, depression and quality of life were significantly correlated with exercise. The variance in several psychosocial factors (benefit finding, medical coping modes, the communication with doctors, depression and quality of life) could be explained by exercise. Psychosocial factors should be addressed and examined over time when evaluating the effect of physical exercise that is prescribed as a clinically relevant treatment.

## Introduction

### Life style behaviors and cancer burden

Cancer is the second leading cause of morbidity and mortality worldwide, with 8 million new cancer cases and 5.3 million cancer-related deaths in economically developing countries, in 2012[1]. Moreover, it is estimated that the future cancer burden will be even greater in these developing countries and that this will be compounded by unhealthy lifestyle behaviors (e.g., smoking, poor diet and physical inactivity), and low economic resources for secondary and tertiary care[2].

### Exercise and cancer prevention

To prevent a proportion of cancers, behavioral interventions may be the most cost-effective long-term cancer control options[3]. In developing countries, physical activity may be a potentially modifiable strategy to reduce cancer risk. The STEPwise approach developed by the World Health Organization (http://www.who.int/chp/steps/instrument/en/), focused on chronic disease risk factor surveillance (STEPS), provides physical activity recommendations and identifies the difficulties associated with in translating knowledge into action[4]. Additional insights should be focus on understanding the underlying mechanisms of the relationship between physical activity and carcinogenesis, which will further aid in identifying molecular targets for intervention[2]. More importantly, a high research priority should be translating knowledge into action by motivating both healthy individuals and cancer survivors to follow the current physical activity recommendations. In particular, shortly after diagnosis, physical activity tends to decline in many cancer patients, and, consequently, most survivors are insufficiently physically active[5].

### Exercise in cancer patients

There have been numerous studies reporting on the association between physical activity and psycho-social factors in cancer populations[6–12]. Findings from previous studies have suggested that highly active individuals were significantly more optimistic than inactive/low-activity individuals[6, 7]. Moreover, several Cochrane systematic reviews have addressed the beneficial effects of exercise on health-related quality of life (QoL) (including decreased depression) in patients with various cancers, both during and after completion anticancer therapy[8, 9]. However, the beneficial effect of exercise on depression may not be significant. Furmaniak and his colleagues showed that exercise may result in less fatigue but little or no difference in depression[10]. Other findings have suggested that establishing support groups, as part of psychosocial rehabilitation services, may help to reduce stress and promote an active lifestyle[11]. After all, exercise has been identified as a modifiable lifestyle factor that is associated with benefit finding (BF) in recent studies[12]. However, these studies primarily focused on the effect of BF on exercise. Moreover, the results were generated from individuals with a specific type of cancer or in young people.

Therefore, while some correlates that were identified via a self-report instrument corresponded with exercise separately, questions remain regarding whether medical coping modes are influenced by physical exercise. There are too few cancer studies with assessments for us to be able to draw firm conclusions. Moreover, there is little research on the relationship between psychical exercise and communication with doctors. Thus, we sought to identify demographic, clinical, and psychosocial correlates of objectively assessed physical exercise in a group of multiple cancer patients as comprehensively as possible, to facilitate the identification and implementation of effective and targeted interventions to improve physical exercise. We tested the following hypothesis in our study: Benefit finding, optimism, medical coping modes, communication with doctors, social support, depression and quality of life are significantly correlated with physical exercise.

## Materials and Methods

### Sample and recruitment

The current study had an observational cross-sectional design. A sample of consecutive patients with early-stage cancer was recruited from the Affiliated Jiangyin Hospital of Southeast University Medical College and the Nantong Tumor Hospital. This study protocol was developed in accordance with our previously published study. Both studies were approved by the institutional review boards of the two hospitals. The Ethics Committee of Clinical Research (ECCR) in Jiangyin Hospital Affiliated to Medical College of Southeast University and the Ethics Committee of Clinical Research (ECCR) in Nantong Tumor Hospital approved the study protocol in accordance with the ethical standards delineated in the Declaration of Helsinki. One hundred and fifty patients with early-stage cancer were recruited from March 2014 to June 2016 from the Affiliated Jiangyin Hospital of Southeast University Medical College. Two hundreds and one patients were recruited from July 2014 to February 2015 from the Nantong Tumor Hospital. All participants fulfilled criteria set in a previous study[13]. All participants provided their written informed consent to participate in this study, and this consent procedure was approved by the ethics committees.

### Data collection

Based on their medical records and mental state, all subjects who were admitted to or attended clinics within the data collection period were eligible to participate in the study. Participants were recruited at clinical oncology clinics during their follow-up appointments. Additionally, hospitalized patients who were awaiting treatment in wards were recruited. These patients were informed of the purpose and nature of the study, expected duration of their participation, anticipated risks/benefits, and voluntary nature of their participation before being invited to participate in the study by the investigators. After signing an informed consent form, participants were invited to complete the study questionnaire either independently or with the assistance of the investigators in a face-to-face interview, which lasted approximately 30 minutes.

Eight instruments were used in the data collection process. (1) Exercise frequency was self-reported and measured by only one question: “How many times do you exercise per week”. The item was scored on the following scale: 1 (rarely exercise), 2 (once a week), 3 (two or three times a week) or 4 (more than 3 times a week). (2) A questionnaire was used to collect socio-demographic and clinical symptom data. This questionnaire was customized and mainly aimed at collecting basic patient information. (3)Psychosocial factors: **a)** Benefit finding was assessed using the Chinese Benefit Finding Scale (CBFS), a six-dimensional, self-report inventory designed and validated in cohorts receiving medical care, specifically individuals with cancer. Each of the 22 items is measured on a five-point Likert scale, with responses ranging from 1 (not at all) to 5 (extremely). With regard to internal consistency, Cronbach’s α of previous BFS scales with various cancer survivors ranged from 0.91 to 0.96[13, 14]. **b)** Communication with doctors was measured using the Communication with Physicians questionnaire which was developed by the Stanford Patient Education Research Center[15]. The instrument has 3 items on a six-point Likert scale ranging from 0 (never) to 5 (always). The content validity of the Chinese version is 0.73, and the test-retest reliability is 0.89. **c)** Developed by Freifel, the 19-item Medical Coping Modes Questionnaire- Chinese version (MCMQ-C) has been widely used to assess the coping modes of patients in relation to a “specific” disease[16]. The original scale was translated and modified by Chinese researchers, with three domains (confrontation, avoidance, resignation) and satisfactory psychometric properties[17]. **d)** Social support was measured by the Social Support Rating Scale (SSRS) developed by Xiao. It is a ten-item instrument that employs a four-point Likert scale (except items 5–7) and has been widely accepted by Chinese researchers[18]. **e)** Depression was measured by the Hospital Depression Scale, which has 7 items on a four-point Likert scale[19]. **f)** The Revised Life Orientation Test (LOT-R) was used to measure the optimism of the participants. It was originally revised by Scheier et al[20] and was later translated into Chinese by Laj et al[21]. The translated scale has 6 items on a five-point Likert scale and has adequate predictive and discriminant validity. (4) Health related quality of life was assessed using the Functional Assessment of Cancer Therapy-General (FACT-G). The original version was developed by Cella et al. and has been widely validated and translated into more than 30 languages[22].

### Data analysis

SPSS-version 17.0 software was adopted to analyze the data. The following descriptive statistics were used to present the demographic data of the participants: means ± SD for normally distributed variables, and frequencies (%) for categorical variables. We analyzed the data using the Pearson Chi-square test and one-way ANOVA to identify variables that were related to exercise. The correlations between exercise and the psychosocial factors and quality of life were calculated using Pearson’s correlation coefficients. The statistical significance level was set at a two-sided p **<** 0.05. Additional multiple regression analyses were used to evaluate the association between exercise and the aforementioned variables.

## Results

### Participant characteristics

Patient demographic and socioeconomic characteristics were reported in our earlier article[13]. A total of 351 subjects (184 men; 52.4%) completed the entire study. The mean age of the study subjects was 57.34 years (SD = 9.05). The average time since cancer diagnosis was 18.94 weeks (SD = 16.88). As shown in Table 1, no difference was found in the subjects’ self-reported exercise with different frequency per week by age (*χ*^2^ = 1.892; p = 0.131) or time since cancer diagnosis (*χ*^2^ = 0.640; p = 0.590). In cancer patients with different occupation types (*χ*^2^ = 14.065; p = 0.029), individual monthly income levels (*χ*^2^ = 24.795; p = 0.003), BMIs (*χ*^2^ = 15.709; p = 0.015) and diagnoses (*χ*^2^ = 42.442; p **<** 0.000), their self-reported exercise frequency per week differed by at least one percentage point.

**Table 1:** Characteristics of participants.

Characteristics	Exercise									
	1		2		3		4		Pearson Chi-square test	p
	n	%	n	%	n	%	n	%		
**Gender**									7.447	0.059
Female	6	46.2	41	52.6	86	52.1	34	35.8		
Male	7	53.8	37	47.4	79	47.9	61	64.2		
**Religious**									1.615	0.656
No	9	69.2	61	78.2	135	81.8	74	77.9		
Yes	4	30.8	17	21.8	30	18.2	21	22.1		
**Occupation**									14.065	**0.029**
Worker	2	15.4	39	50.0	81	49.1	43	45.3		
Farmer	6	46.2	28	35.9	70	42.4	36	37.9		
Other	5	38.4	11	14.1	14	8.5	16	16.8		
**Monthly individual income**									24.795	**0.003**
<¥1000	2	15.4	19	24.4	36	21.8	26	27.4		
¥1000-¥2000	4	30.8	35	44.9	90	54.5	39	47.9		
¥2000-¥5000	6	46.2	19	24.4	38	23.0	18	23.1		
>¥5000	1	7.7	5	6.4	1	0.6	12	5.4		
**BMI (kg/m**^**2**^**)**									15.709	**0.015**
<18.5	3	23.1	12	15.4	22	13.3	16	16.8		
18.5–25	5	38.5	55	70.5	118	71.5	51	53.7		
>25	5	38.5	11	14.1	25	15.2	28	29.5		
**Diagnosis**									42.442	**< 0.000**
Breast cancer	4	30.8	28	35.9	67	40.6	12	12.6		
Colorectal cancer	3	30.8	17	21.8	34	20.6	28	29.5		
Gastric cancer	3	23.1	11	14.1	33	20.0	25	26.3		
Liver cancer	1	7.7	11	14.1	10	6.1	11	11.6		
Esophageal cancer	1	7.7	3	3.8	17	10.3	17	17.9		
Other	1	7.7	8	10.3	4	2.4	2	2.1		

### Psychosocial factors and health-related QoL

The mean and standard deviation of benefit finding, communication with doctors, medical coping modes, social support, depression, optimism, and quality of life are presented in Table 2. Differences in the frequency of exercise were associated with different degrees of reported BF (F = 24.651; p < 0.000), communication with doctors (F = 15.285; p **<** 0.000), medical coping modes (F = 45.912; p < 0.000), social support (F = 2.938; p = 0.030), depression (F = 6.017; p < 0.000), and quality of life (F = 12.288; p < 0.000). In addition, the Pearson’s correlation coefficients for psychosocial factors in early-stage colorectal cancer patients are shown in Table 3. Significant associations were found between exercise and the following BF (r = 0.251), communication with doctors (r = 0.175), medical coping modes (r = 0.253), depression (r = -0.128) and quality of life (r = 0.116). However, no significant associations were found between exercise and social support (r = -0.003) and optimism (r = 0.027).

**Table 2:** Participant characteristics.

Measured factors	Exercise										
	1		2		3		4		F	p
	$\bar{X}$	SD	$\bar{X}$	SD	$\bar{X}$	SD	$\bar{X}$	SD		
Benefit finding	75.15	9.83	75.19	10.99	78.05	9.10	82.57	11.17	24.651	**< 0.000**	
Communication with doctors	5.92	2.98	7.14	1.98	7.04	2.00	7.94	2.38	15.285	**< 0.000**	
Medical coping modes	42.92	4.79	43.18	4.36	42.36	4.09	45.74	4.62	45.912	**< 0.000**	
Social support	45.92	3.03	44.24	3.45	45.17	3.70	44.64	4.39	2.983	**0.030**	
Depression	9.38	1.80	8.95	1.87	8.53	1.70	8.28	1.93	6.017	**< 0.000**	
Optimism	16.62	1.56	17.01	2.36	17.50	2.16	17.33	2.32	1.805	0.145	
Quality of life	82.04	10.02	88.51	8.24	91.10	5.78	90.69	7.45	12.288	**< 0.000**	

**Table 3:** Exercise as a predictor of health and other factors in multiple regression models.

Outcome variables	Exercise			
	R^2^	β	p	Pearson’s r
Benefit finding	0.063	0.227	**< 0.000**	0.251*
Communication with doctors	0.028	0.170	**< 0.000**	0.175*
Medical coping	0.064	0.233	**< 0.000**	0.253*
Social support	0.001	0.025	0.402	-0.003
Depression	0.016	-0.129	**< 0.000**	-0.128*
Optimism	0.001	0.027	0.964	0.027
Quality of life	0.018	0.115	**< 0.000**	0.116*

* p **<** 0.05

### Exercise as a predictor of psychosocial factors and QoL

Multiple regression analyses were used to evaluate the association between exercise and benefit finding, communication with doctors, medical coping modes, social support, depression, optimism and quality of life, after controlling for occupation type, individual monthly income level, BMI and diagnosis. As demonstrated in Table 3, exercise explained 1.6%-6.4% of the variance in five variables, excluding social support and optimism.

## Discussion

The current study examined possible health-related quality of life, demographic, clinical and psychosocial correlates of exercise in early-stage cancer patients. We found that demographic and clinical factors, occupation type, individual monthly income level, BMI and diagnosis were significantly different in cancer patients who reported different exercise frequencies. Other psychosocial factors, including benefit finding, medical coping modes, communication with doctors, social support, depression and quality of life were significantly correlated with exercise. When controlling for the demographic and clinical factors, exercise could separately explain several of psychosocial factors (benefit finding, medical coping modes, the communication with doctors, depression and quality of life).

Our finding that BMI was significantly associated with physical exercise is in line with previous studies[23,24] of various cancer populations employing self-reported physical activity questionnaires. Determining the causal direction of the association between physical exercise and BMI was restricted in this current study by the cross-sectional design. Diagnosis was also found to be significantly associated with physical exercise. This factor could highlight those subpopulations that are more likely to be physically inactive (e.g., colorectal cancer patients) and thus may have a great need for healthy life style changes. Other demographic factors, including occupation type and monthly individual income level, were correlated with physical exercise. The physical activity associated with different occupations and different exercise options during leisure time was primarily affected by income level. With detailed insight into the mechanistic effects of exercise on tumor biology and growth, there would be a strong rationale for prescribing effective exercise interventions for cancer patients would be provided[25]. Nevertheless, our study indicates that different exercise frequencies are related to demographic and clinical factors, thereby providing information that allows exercise to be prescribed and effects to be observed in the appropriate groups.

We found that a higher frequency of physical exercise was significantly associated with a higher benefit finding score. This finding is consistent with previous studies on cancer survivors[13, 25]. One explanation for this result is that physical exercise as a coping strategy can release and alleviate psychological pressure, to help patients to adjust psychologically to cancer and improve their sense of BF, through enhancing their self-esteem[26]. As a result, interventions aimed at encouraging cancer survivors exercise at a proper frequency regularly may be more effective when including strategies to increase benefit finding in these populations, especially for those who would not be likely to undertake new activities and stay engaged.

As we hypothesized earlier, depression was correlated with physical exercise in our study. Ruas and colleagues proposed a mechanistic link between exercise training and reduced depression and found that exercise training could decreased kynurenine levels through increasing kynurenine metabolism in the muscles[27]. In this sense, physical exercise could be an effective intervention for cancer patients with depression.

Our investigation was conducted in a hospital setting; therefore, doctors were part of the patients’ social support system throughout their illness experience. Thus, communication with doctors was selected as an influential factor to included in the study. According to cognitive adaptation theory [28], cancer patients can gain control of their health through regular exercise, and to achieve this, patients might need a more scientifically based exercise ‘prescription’ from a doctor. As a result, the variance in both medical coping modes and communication with doctors was found to be explained by physical exercise. Hence, smooth and frequent communication with doctors is needed and medical coping modes will tend to be confrontatioal in patients who enjoy this frequent communication with doctors.

With regard to social support, cancer patients exercised at different frequencies based on their levels of social support in our study. Given the subtle relationship between social support and exercise, exercise could expand individuals’ social circle and help them to gain the support of new friends. However, there was no correlation between exercise and social support, as personality traits would have a great effect. Therefore, exercise is not sufficient to provide patients with more social support.

In contrast to our hypothesis, optimism was not associated with physical exercise. Previous research showed that dispositional optimism was not only correlated positively with problem-focused coping but also associated with a faster rate of physical recovery and a faster rate of return to normal life activities subsequent to discharge[29]. Optimism is a type of positive personality traits that is not easily changed. More specifically, it could probably be the reason why physical exercise could not explain the variance in optimism in our study. Other reasons relate to the participants in our study, including their relatively short experience of cancer (less than 2 years), and the epidemiological differences between the patients with different types of cancer.

The current study had several limitations. First, participants were included based on convenience sampling of cancer patients in Nantong and Jiangyin, which potentially limits the generalizability of the results to patients living in other Chinese provinces. Second, self-reported and closed-ended questionnaires were the only instruments employed in the study. Third, there was a small sample size in this study. Fourth, the intensity of the exercise was not examined in this study. It could have been measured by heart rate and self-reported feelings. This study should be regarded as preliminary and hence, further studies are needed to address these limitations.

## Conclusions

In conclusion, our results showed that benefit finding, medical coping modes, communication with doctors, social support, depression and quality of life are significantly correlated with exercise. Physical exercise plays an important role in explaining several of psychosocial factors (benefit finding, medical coping modes, communication with doctors, depression and quality of life). Therefore, when recommending the prescription of physical exercise as a supportive self-care intervention or a clinically relevant treatment, psychosocial factors should be addressed and examined over time when evaluating any effects.

## Supporting Information

S1 File EpiData 315. sav. The data set of this study.
(SAV)
